# Defining syndromes using cattle meat inspection data for syndromic surveillance purposes: a statistical approach with the 2005–2010 data from ten French slaughterhouses

**DOI:** 10.1186/1746-6148-9-88

**Published:** 2013-04-30

**Authors:** Céline Dupuy, Eric Morignat, Xavier Maugey, Jean-Luc Vinard, Pascal Hendrikx, Christian Ducrot, Didier Calavas, Emilie Gay

**Affiliations:** 1 Unité Epidémiologie, Agence nationale de sécurité sanitaire de l’alimentation, de l’environnement et du travail (Anses), 31 avenue Tony Garnier, Lyon, Cedex 07, F69364, France; 2 Unité d’épidémiologie animale, UR346, INRA, St Genès Champanelle, 63122, France; 3 Direction générale de l’alimentation, 251, rue de Vaugirard, Paris, Cedex 15, 75732, France; 4 Direction scientifique des laboratoires, Agence nationale de sécurité sanitaire de l’alimentation de l’environnement et du travail (Anses), 37-31 avenue du général, Maisons-Alfort, Cedex, Leclerc F-94701, France

**Keywords:** Syndromic surveillance, Animal health, Meat inspection, Slaughterhouses, Cattle

## Abstract

**Background:**

The slaughterhouse is a central processing point for food animals and thus a source of both demographic data (age, breed, sex) and health-related data (reason for condemnation and condemned portions) that are not available through other sources. Using these data for syndromic surveillance is therefore tempting. However many possible reasons for condemnation and condemned portions exist, making the definition of relevant syndromes challenging.

The objective of this study was to determine a typology of cattle with at least one portion of the carcass condemned in order to define syndromes. Multiple factor analysis (MFA) in combination with clustering methods was performed using both health-related data and demographic data.

**Results:**

Analyses were performed on 381,186 cattle with at least one portion of the carcass condemned among the 1,937,917 cattle slaughtered in ten French abattoirs. Results of the MFA and clustering methods led to 12 clusters considered as stable according to year of slaughter and slaughterhouse. One cluster was specific to a disease of public health importance (cysticercosis). Two clusters were linked to the slaughtering process (fecal contamination of heart or lungs and deterioration lesions). Two clusters respectively characterized by chronic liver lesions and chronic peritonitis could be linked to diseases of economic importance to farmers. Three clusters could be linked respectively to reticulo-pericarditis, fatty liver syndrome and farmer’s lung syndrome, which are related to both diseases of economic importance to farmers and herd management issues. Three clusters respectively characterized by arthritis, myopathy and Dark Firm Dry (DFD) meat could notably be linked to animal welfare issues. Finally, one cluster, characterized by bronchopneumonia, could be linked to both animal health and herd management issues.

**Conclusion:**

The statistical approach of combining multiple factor analysis with cluster analysis showed its relevance for the detection of syndromes using available large and complex slaughterhouse data. The advantages of this statistical approach are to i) define groups of reasons for condemnation based on meat inspection data, ii) help grouping reasons for condemnation among a list of various possible reasons for condemnation for which a consensus among experts could be difficult to reach, iii) assign each animal to a single syndrome which allows the detection of changes in trends of syndromes to detect unusual patterns in known diseases and emergence of new diseases.

## Background

The main goal of meat inspection is to guarantee food safety. An ante-mortem and post-mortem inspection of each animal slaughtered in a European country is performed by veterinary services to detect signs or lesions that can lead to the condemnation of offal, part of the carcass or the whole carcass if there is a danger for human consumption or an organoleptic quality problem [1]. Considering this goal, data collected in slaughterhouses are mainly pre-diagnostic and non-specific (except for notifiable diseases such as tuberculosis). These data, related to diseases and other disorders, not available elsewhere, can be registered in real time during the slaughtering process as demonstrated by pilot systems such as the *Nergal-Abattoir* system in France [2]. This system has been developed by the French Ministry of Agriculture in ten cattle slaughterhouses and it was used to collect data in real time during the slaughtering process. Both demographic data (sex, age, breed) and health related data (reasons for condemnation, condemned portions) were collected for each slaughtered animal. The large amount of data available from the *Nergal-Abattoir* system, nearly 2 million cattle, might be the basis of a syndromic surveillance system in France, based on meat inspection data.

Indeed, syndromic surveillance is defined as the monitoring of non-specific health indicators including clinical signs, symptoms or proxy measures to enable the early identification of the impact (or absence of impact) of potential human or veterinary public health threats. It is implicit that the data are usually collected for purposes other than surveillance and, if possible, are automatically generated so as not to impose an additional burden on the data providers [3]. Slaughterhouse data could then seem relevant for syndromic surveillance, as a complement to other existing animal health surveillance systems.

In classical epidemiological surveillance, objectives are defined and relevant data are then collected to meet these objectives. In syndromic surveillance, available data, usually collected for another purpose, are used for an epidemiological surveillance objective without being able to have an impact on the way they are collected. The procedure to define a case is thus inevitably different than for classical surveillance. According to the type of meat inspection data used, different kinds of epidemiological surveillance could then be performed. Specific surveillance (i.e. surveillance with a targeted objective) is focused on the surveillance of a pre-defined disease or group of diseases whereas non-specific surveillance (i.e. surveillance with a non-targeted objective) aims at detecting unknown or emergent diseases [4]. Syndromic surveillance can be either specific or non-specific according to the nature of the indicator monitored.

Meat inspection generates a huge amount of data that are rarely used for animal health and welfare surveillance purposes. Studies were recently published using these data for syndromic surveillance, including Alton et al. [5,6] who conducted a risk factor analysis to study the suitability of cattle condemnation data for syndromic surveillance in Ontario slaughterhouses.

There are many different reasons for condemnation and condemnation portions that could be more or less frequent according to demographic aspects (age, sex, production type). The first difficulty is thus to determine which reason for condemnation or group of reasons for condemnation linked to food safety could define a relevant animal health or animal welfare indicator for a specific or non-specific surveillance system. To deal with this issue of surveillance indicator, this paper proposes an innovative statistical approach to evidence a typology of cattle that had at least one portion of the carcass condemned. Multiple factor analysis (MFA) in combination with clustering methods was thus performed on meat inspection data available from the *Nergal-Abattoir* French system to identify which lesions or groups of lesions could be used as indicators for specific or non-specific syndromic surveillance.

## Methods

### Materials

In European countries, each slaughtered animal is submitted to ante and post-mortem inspection so as to guarantee food safety. From 2005 to 2010, the French Ministry of Agriculture started the *Nergal-Abattoir* project to collect data in real time during the slaughtering process. It involved ten cattle slaughterhouses in France that represented about 20% of cattle slaughtered in the country. Data were collected using touch screens on the slaughter lines, provided by the food business operator. Data were then transmitted through a constant data flow to the database of the French Ministry of Agriculture. The main objectives of this system were to guarantee the traceability of meat inspection results (quality assurance) and to automatically produce the mandatory condemnation reports for cattle owners.

For each animal, the database contained: identification number, date of birth and slaughter, last farm location, sex, breed, signs observed during ante-mortem inspection, reasons for condemnation and locations or absence of condemnation.

From June 2005 to December 2010, 1,937,980 cattle were slaughtered in the ten slaughterhouses involved in the *Nergal-Abattoir* project. Cattle with missing data (n=63) were excluded. Cattle euthanized or that died in the ante-mortem inspection area, respectively 1,186 and 353 animals, were excluded from this study. Among the population of 1,937,917 cattle slaughtered without missing data, 381,186 had at least one part of the carcass condemned.

The data available for each slaughterhouse did not cover the same period because the *Nergal-Abattoir* project did not start and finish at the same dates for each slaughterhouse, thus the number of days of available data varied from 345 to 1,698 days.

**Figure 1:**
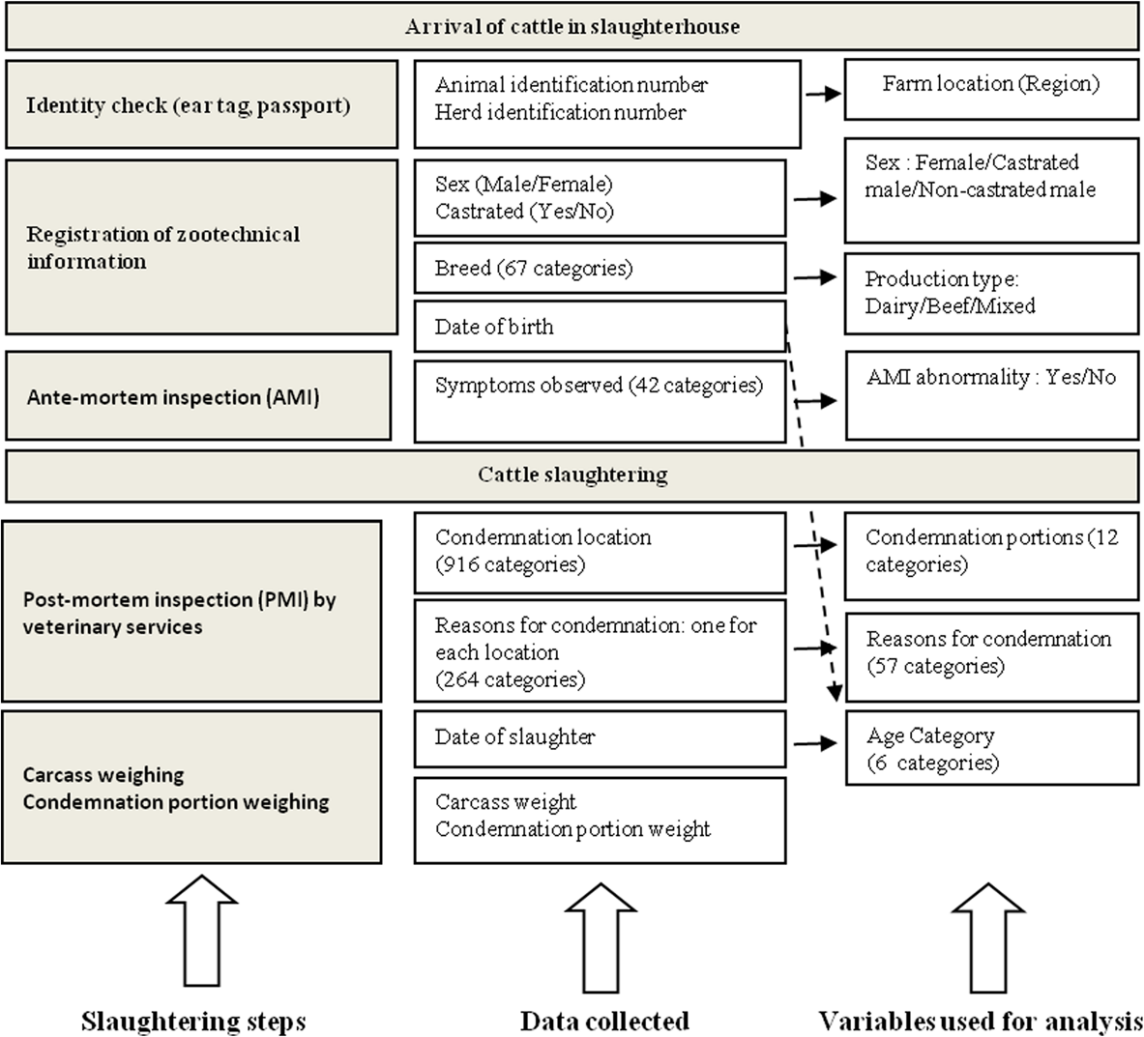
Different steps in the slaughtering process, data collected and variables created for analysis.

All data registered during the slaughtering process and used to create categorical variables for analyses are presented in Figure 1.

Some levels of the categorical variables were grouped to avoid low numbers in levels that could create instability in data analyses (Figure 1). The location of the farm was used to allocate each animal to a region. Regions with a frequency lower than 1% (among condemned animals) were grouped together in an “Other regions” level. The 67 breeds, available in the database, were grouped according to production type as defined by FranceAgriMer, i.e. French national organization of agriculture products, [7] into the levels “dairy”, “beef” and “mixed cattle”. Age categories were built according to the fact that i) management practices are different according to age categories, ii) European regulation defines a specific age category for veal (animal under 8 months of age) [8], iii) a French observatory of livestock mortality already defined age categories in line with management practices [9]. The ages of cattle were thus classified into six levels: [0;8 months old), [8;24 months old), [2;3.5 years old), [3.5;5 years old), [5;10 years old), ≥10 years old.

Clinical signs observed during ante-mortem inspection were used as binary variables: presence/absence. The list of reasons for condemnation is a national mandatory list in France [10].

**Figure 2:**
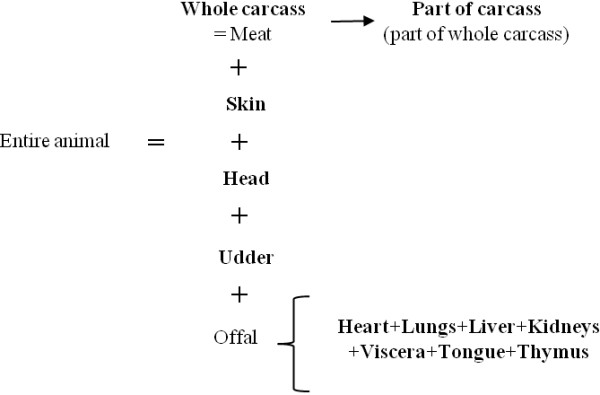
Presentation of the 12 different portions (in bold font) of cattle carcass.

Because of the database design, each condemned portion was associated with one and only one reason for condemnation. As each animal could have more than one condemned carcass portion, it could also have more than one reason for condemnation (*e.g.* condemnation of the liver for abscess and the heart for pericarditis). The 264 different reasons for condemnation of the *Nergal-Abattoir* system were merged into 57 reasons for condemnation levels according to their biological similarities or in order to compare data among slaughterhouses when the levels of detail of the reasons for condemnation were different (*e.g.* “abscess”, “multiple abscess”, “local abscess” were merged into “abscess”). The condemned portions were merged into 12 levels (Figure 2).

### Method

A principal component method, Multiple Factor analysis (MFA), in combination with clustering methods (K-means and Hierarchical Ascendant Clustering) was used to establish groups of condemned cattle, i.e. cattle with at least one portion of the carcass condemned. Calculations were performed with R software [11]. Additional information on the statistical methods used is presented in Additional file 1.

#### Multiple factor analysis

To perform clustering methods, the distance between two units needs to be defined. Using a principal component method as a first step allowed the computation of the Euclidean distance between units i.e. condemned cattle. We wanted to compute a global distance between condemned cattle based on both demographic and condemnation data and to balance the influence of these two sets of variables on this computation. Multiple Factor Analysis was the suitable principal component method to achieve these two objectives.

The principle of this method is to reduce multidimensional data to their principal components, based on the assumption that the studied variables are not independent of each other [12,13]. Each animal is represented in a space with factorial axes defined by the best linear combination of the active variables, i.e. observed variables. Factorial axes are constructed from active variables whereas the result interpretation is aided by supplementary variables. The supplementary variables are projected onto the vector subspace generated by the factors. The particularity of MFA is to compute a distance between individuals corresponding to a weighted sum of the separate distances induced by every set of variables. The contribution of any set of variables to the global distance depends on the dimension of the unit cloud defined by the separate Multiple Component Analysis of each set of variables i.e. a cloud with several important orthogonal variance directions has a greater influence than a one-dimensional cloud [12,14].

The two groups of active variables were demographic variables (sex, age category, production type) and condemnation data (reasons for condemnation and portions). Only levels of reasons for condemnation and levels of condemned portions with percentages higher than 1% were used as active variables to avoid instability in the MFA [15].

Six supplementary variables were used: year of slaughter, month of slaughter, farm location, presence or absence of clinical signs during ante-mortem inspection, abattoir identification number, reasons for condemnation and portions with frequencies lower than 1%. MFA was performed with the “FactoMineR” R package [16].

#### Hybrid clustering: K-means and hierarchical ascendant clustering

Clustering of cattle characteristics was investigated using the Euclidian distance between principle coordinates [12]. Principal coordinates were determined in a subspace that ensured good quality representation of the data to limit noise, i.e. the number of factorial axes ensuring 95% of the total variance were considered [13,15].

HAC was the appropriate clustering method for operating from coordinates issued from a principal component method and to achieve our objective [12,14,17]. However, considering the large amount of data (381,186 cattle), it was not possible to directly perform a hierarchical ascendant clustering (HAC), which requires access to computers with extremely high computational and storage capacity, on the principal coordinates provided by MFA. Therefore hybrid clustering, i.e. combining several clustering methods to take advantage of their specific strengths, was performed using K-means, known for its efficiency for clustering large datasets, as a first step for HAC [18,19]. K-means clustering was performed on the MFA principal coordinates. The number of clusters was defined as the number of distinct principal coordinates, because similarities among principal coordinates showed that a significant number of cattle had the same principal coordinates. The number of clusters defined was then small enough to perform HAC on the centroids of the clusters. The generalized Ward’s criterion was used as the aggregation criterion for HAC. It consists in aggregating two clusters in a way that minimizes intra-cluster variance and maximizes inter-cluster variance [20]. The partition was determined considering the hierarchical tree and according to the biological meaning of the clusters [15].

HAC was consolidated by a K-means performed on the centers of the HAC clusters. HAC and K-means were performed respectively with R packages “cluster” and “stats” [11].

#### Description of clusters

Description and interpretation of the clusters were based on levels of both active and supplementary variables, using the V-test value to decide which levels had to be kept for the description of each cluster [15,16]. V-test values measure the distance, for each variable level, between the within-group proportion and the overall proportion formulated by a number of standard deviations of Gaussian law: a value of the V-test greater than 1.96 corresponds to a p-value less than 0.05. Thus, the higher the difference between these two proportions, the higher the absolute value of the V-test. Variable levels with the highest absolute V-test values were considered to characterize a cluster in comparison to the whole population of condemned cattle. To identify these variable levels, a histogram of ordered absolute V-test values was used for each cluster to find the point of changing slope i.e. point that defined the limit of the V-test values considered as highest. The variable levels identified with this process for each cluster were then used to describe the cluster using both proportion of cattle with the variable level within the cluster and the proportion of cattle with the variable level that were in this cluster. The description of the clusters was performed using the “FactoMineR” R package [16].

We created indicators to quantify the stability of the clusters. The objective of these descriptive indicators was to identify which clusters, i.e. groups of reasons for condemnation, were commonly seen in slaughterhouses and which ones were more specific to some slaughterhouses or periods of time. The stability was evaluated by year of slaughter and by slaughterhouse through the same process, i.e. MFA, K-means, and HAC consolidated by K-means, as part of the objective to evaluate whether the partition was impacted by the year of slaughter or the slaughterhouse practices. Stability was defined through three indicators 1) the number of slaughterhouses for which the cluster has been identified; 2) the number of years of slaughter for which the cluster has been identified; 3) the addition of the two previous indicators. For this last indicator a cluster identified for example in seven out of the ten slaughterhouses and four out of the five years of slaughter had ((7+4)/(10+5))*100= 73% of stability. Clusters with a value higher than 50% for this latter indicator were considered as stable in this study.

Interpretation of the clusters was based on i) the statistical description of the cluster using V-tests, ii) a literature review to determine which condition or infection could be linked to the reasons for condemnation that characteristized each cluster, iii) meat inspection expert opinion on the interpretation of clusters and possible use in defining syndromes for syndromic surveillance.

The opinion of experts was obtained through an already existing French group of around twenty meat inspection experts that consisted of veterinary school professors, veterinary meat inspectors and national meat inspection referees. A presentation of the methodology and statistical results of this study was conducted during a dedicated meeting. It was followed by a discussion among experts to validate the biological meaning of each group of lesions and discuss their interpretation.

## Results

### Descriptive statistics

Depending on the slaughterhouse, the mean number of cattle slaughtered each day varied from 122 to 543. The proportion of cattle with at least one condemned portion varied from 10% to 36% (Table 1).

**Table 1:** **Description of data available in the *Nergal-Abattoir *project database in the ten slaughterhouses involved**

**Slaughter-house**	**First day of data availability**	**Last day of data availability**	**Number of days of data availability**	**Mean number of cattle slaughtered each day**	**Total number of cattle slaughtered**	**Total number of condemned cattle**^**1**^	**Percentage of condemned cattle**^**1 **^**(%)**
1	2006-05-12	2010-12-31	1,698	305	352,997	57,355	16.25
2	2006-06-19	2010-03-25	1,375	275	261,002	75,095	28.77
3	2006-11-23	2010-12-30	1,498	122	126,757	30,204	23.83
4	2006-10-12	2009-05-11	942	235	149,823	14,317	9.56
5	2007-10-08	2008-09-17	345	137	30,624	3,442	11.24
6	2005-06-02	2009-10-09	1,590	543	598,813	86,109	14.38
7	2007-03-06	2010-09-17	1,291	133	95,291	26,295	27.59
8	2007-03-27	2008-09-18	541	142	52,446	18,848	35.94
9	2007-06-26	2010-01-19	938	254	164,038	49,684	30.29
10	2006-03-15	2008-02-01	688	224	106,126	19,837	18.69
Total	-	-	-	-	1,937,917	381,186	19.67

^1^ Condemned cattle= cattle with at least one portion of the carcass condemned.

#### Active variables

Among the 381,186 cattle included in the study, 70% were females, 26% non-castrated males and 4% castrated males. Beef cattle represented 44% of the cattle condemned, dairy cattle 35%, and mixed cattle 20%. Most of the cattle condemned belonged to the 5-to-10 year old age category (37%) (Table 2).

**Table 2:** Description of condemned cattle according to sex, production type and age category

		**Number of condemned cattle**^**1**^	**Percentage of condemnations (%)**
Sex	Female	266,795	69.99
	Castrated male	17,075	4.48
	Non-castrated male	97,316	25.53
Production type	Dairy	134,445	35.27
	Mixed	77,999	20.46
	Beef	168,742	44.27
Age category	[0;8 months old)	8,475	2.22
	[8;24 months old)	77,370	20.3
	[2;3.5 years old)	50,545	13.26
	[3.5;5 years old)	52,406	13.75
	[5;10 years old)	142,462	37.37
	≥10 years old	49,928	13.1

^1^ Condemned cattle= cattle with at least one portion of the carcass condemned.

The mean number of different condemned portions per animal was 1.8 (681,163 condemnation portions for 381,186 cattle condemned) with a minimum of one and a maximum of 18. Overall 80% of the cattle condemned had only one portion of the carcass condemned.

The description of the 12 listed portions showed that 68% of the cattle with at least one condemned portion were related to condemnation of the liver and 3% of condemnations involved the whole carcass being condemned (Table 3).

**Table 3:** Number and proportion of cattle concerned by condemned portions and reasons for condemnation

	**Number of animals**	**Percentage of condemnations (%)**
**Condemned portion**		
Liver	257,377	67.52
Kidneys	73,310	19.23
Lungs	68,976	18.10
Viscera	57,300	15.03
Part of the carcass	48,841	12.81
Heart	38,515	10.10
Head	29,993	7.87
Udder	20,155	5.29
Tongue	14,728	3.86
Whole carcass	13,074	3.43
**Reason for condemnation**		
Abscess	89,795	18.76
Liver fluke	72,059	15.06
Preventive reason for condemnation^1^	45,719	9.55
Sclerosis	38,621	8.07
Macular telangiectasia	31,639	6.61
Nephritis	29,290	6.12
Other reason for condemnation^2^	19,921	4.16
Cyst	16,951	3.54
Peritonitis	13,753	2.87
Bronchopneumonia	13,442	2.81
Infiltration	13,252	2.77
Arthritis	11,362	2.37
Fecal contamination	11,243	2.35
Steatosis	10,718	2.24
Other deterioration^3^	10,482	2.19
Lung emphysema	8,432	1.76
Pericarditis	8,282	1.73
Myopathy	7,780	1.63
Meat with abnormal maturation	5,558	1.16
Local muscular cysticercosis	5,132	1.07

^1 “^Preventive reason for condemnation” was assigned to carcass portion/organ that was not submitted for a post-mortem inspection or for offal that was condemned on the slaughter line when the carcass and its offal were detained.

^2 “^Other reason for condemnation” is not the result of the merging of existing reasons for condemnation. This was a possible reason for condemnation voluntarily chosen by an official inspector.

^3^ “Other deterioration” included hemorrhagic lesions of lungs or muscles linked to problems in the slaughtering process; superficial and deep putrefaction.

Only reasons for condemnation and portions representing at least 1% of the animals are presented. Each animal can have more than one portion of the carcass condemned but each part of the carcass can be linked to only one reason for condemnation.

Among the 57 reasons for condemnation, 44 were used during the study period. There was an average of 1.3 reasons for condemnation per condemned animal with a maximum of 8. Overall 90% of condemned cattle had at least two different reasons for condemnation. The most frequent reasons for condemnation were “abscess” (19%) and “liver fluke” (15%) (Table 3).

#### Supplementary variables

Reasons for condemnation and portions with percentages lower than 1% were used as supplementary variables. The description of condemned cattle by slaughterhouse identification number is presented in Table 1. The most frequent regions of the last farm location of the condemned cattle were Basse-Normandie and Pays de la Loire in western France (Additional file 2). The variation in the number of condemned cattle according to month and year of slaughter was linked to the difference in the period of data availability for each slaughterhouse (Additional file 2). Among the condemned cattle, 4% presented a clinical sign during ante-mortem inspection.

### Multiple factor analysis and clustering

We used two groups of active variables: the first one contained sex, age category and the three production types; the second contained the condemnation portions and reasons for condemnation.

**Figure 3:**
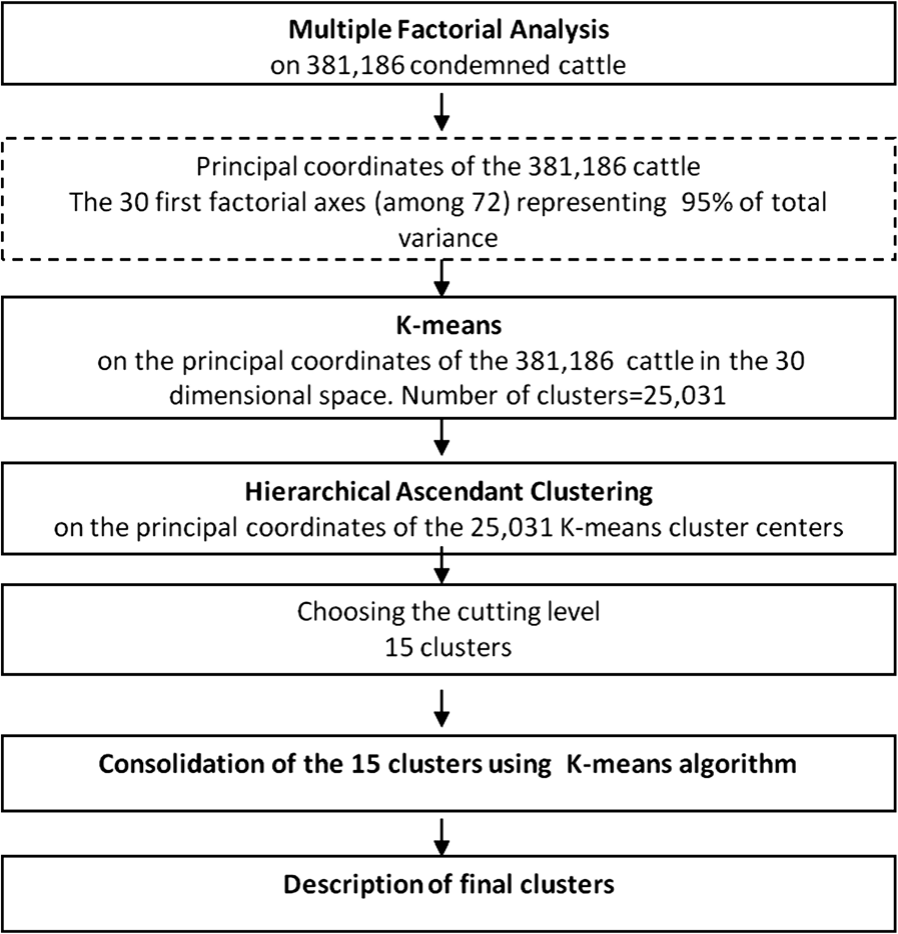
Flow chart of the main steps of the statistical analysis performed to define the typology of condemned cattle.

The first 30 component axes of MFA represented more than 95% of the total variance of the 72-dimensional space. K-means was performed on the 25,031 distinct coordinates in the 30-dimensional space (Figure 3).

The group of demographic variables contributed to 69% of the construction of the first factorial axis of MFA and was almost the only group contributing to the construction of the third axis (96%). The second group of active variables (reasons for condemnation and condemnation portions) greatly contributed to the construction of the second factorial axis of MFA (74%) (Table 4). These observations were confirmed by the high value of correlation between the demographic variables group and the first and third factorial axis (0.88 and 0.99) and between reasons for condemnation and condemnation portions group and the second factorial axis (0.87) (Table 4).

**Table 4:** Contribution and correlation of each group of active variables with each MFA factor

**Group of variables**		**Dim.1**	**Dim.2**	**Dim.3**	**Dim.4**	**Dim.5**
Demogaphic variables	Contribution (%)	69.358	26.407	95.683	76.275	19.871
	Correlation	0.879	0.531	0.988	0.888	0.460
Reason for condemnation and condemnation portions	Contribution (%)	30.642	73.593	4.317	23.725	80.129
	Correlation	0.631	0.868	0.277	0.515	0.902

**Figure 4:**
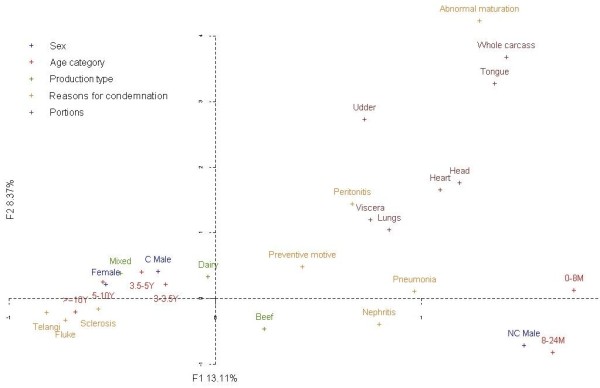
**Representation of the categories of variables equal to 1 in the first factor plan.** Only reasons for condemnation and portions that contributed greatly to the first or second factorial axis are shown. All demographic variables categories are shown**.**

An increasing gradient of age was visible along the first factor of MFA from the right to the left. The first factorial axis placed castrated and non-castrated males on one side and mixed cattle and beef cattle on the other side. The second factor made a separation between abnormal meat maturation and a group of liver lesions, i.e. macular telangiectasia, liver fluke and sclerosis (Figure 4).

HAC was performed on the centers of K-means clusters defined by coordinates in the 30-dimensional-space of the MFA (Figure 3).

**Figure 5:**
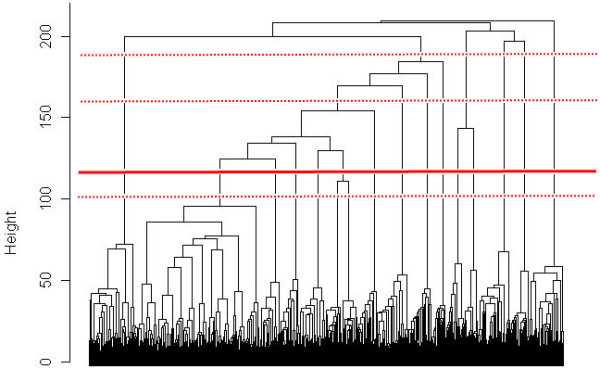
**Dendrogram of the HAC performed on MFA coordinates in 30-dimensional space.** The dotted red lines represent partitions tested and the full red line represents the final partition.

The hierarchical tree suggested four possible partitions into 6, 9, 15 or 16 clusters based on the height of the HAC dendrogram (Figure 5). Based on the biological significance of the clusters of these partitions, the 15 clusters partition was selected. The partition was strengthened by the K-means method and each animal was attributed to its cluster (Figure 3).

The comparison of the proportions of the levels of active and supplementary variables in each cluster and in the whole dataset made it possible to identify which levels described each cluster in the best way. Results of this description are presented in Tables 5 and 6.

**Table 5:** Cluster description by categories of active and supplementary variables (Cluster 1 to 7)

**Cluster**	**Number of condemned animals (%)**	**Variable category**	**Cla/Mod**^**1**^	**Mod/Cla**^**2**^	**Global**^**3**^	**|v.test|**
1	9,268 (2.43%)	Fecal contamination=1	82.43	100	2.95	Inf
		Lungs=1	10.92	81.26	18.10	Inf
		Heart=1	14.79	61.45	10.10	Inf
		Abattoir number=7	6.5	18.44	6.9	37.52
		Other motive=1	6.56	14.10	5.23	32.64
		Age=[8;24 months old)	4.1	34.19	20.30	31.55
		Sex=Non castrated male	3.81	39.95	25.53	30.75
2	7,215 (1.89%)	Bronchopneumonia=1	15.39	28.68	3.53	Inf
		Pericarditis=1	87.12	100	2.17	Inf
		Lungs=1	4.81	45.99	18.10	Inf
		Heart=1	18.68	99.7	10.10	Inf
		Abattoir number=6	3.32	39.58	22.59	32.64
		Production type=Dairy cattle	2.76	51.34	35.27	28.15
		Region=Basse-Normandie	3.00	33.83	21.33	24.74
3	250,256 (65.65%)	Abattoir number=3	85.20	10.28	7.92	Inf
		Abattoir number=2	77.97	23.40	19.70	Inf
		AMI abnormality=No	66.98	98.03	96.09	Inf
		Region=Rhône-Alpes	83.02	6.61	5.23	Inf
		Region=Franche-Comté	82.73	3.70	2.94	Inf
		Region=Burgundy	81.86	6.61	5.30	Inf
		Region=Auvergne	76.63	11.32	9.70	Inf
		Macular telangiectasia=1	87.65	11.08	8.30	Inf
		Liver fluke=1	88.95	25.61	18.90	Inf
		Sclerosis=1	80.97	12.50	10.13	Inf
		Abscess=1	74.52	26.74	23.56	Inf
		Liver=1	76.88	79.07	67.52	Inf
		Production type=Beef cattle	71.97	48.53	44.27	Inf
		Age=≥10 years old	74.72	14.91	13.10	Inf
		Age=[5;10 years old)	71.21	40.54	37.37	Inf
		Sex=Female	71.03	75.73	69.99	Inf
4	8,736 (2.29%)	Abattoir number=6	4.21	41.49	22.59	Inf
		Peritonitis=1	63.52	100	3.61	Inf
		Preventive motive=1	6.01	31.44	11.99	Inf
		Abscess=1	4.18	42.96	23.56	Inf
		Viscera=1	11.96	78.45	15.03	Inf
		Udder=1	7.75	17.89	5.29	Inf
		Part of the carcass=1	9.69	54.17	12.81	Inf
		Region=Basse-Normandie	4.00	37.21	21.33	34.28
5	6,257 (1.64%)	Congestion=1	18.03	7.88	0.72	Inf
		Pleurisy	22.99	8.17	0.58	Inf
		AMI abnormality=Yes	8.75	20.86	3.91	Inf
		Peritonitis=1	16.93	37.22	3.61	Inf
		Whole carcass=1	40.91	85.47	3.43	Inf
		Viscera=1	9.47	86.69	15.03	Inf
		Tongue=1	42.38	99.76	3.86	Inf
		Head=1	2.79	99.68	7.87	Inf
		Kidneys=1	8.38	98.16	19.23	Inf
		Lungs=1	7.93	87.45	18.10	Inf
		Udder=1	21.89	70.50	5.29	Inf
		Liver=1	2.40	98.59	67.52	Inf
		Heart=1	16.15	99.39	10.10	Inf
6	15,538 (4.08%)	Abattoir number=8	13.26	16.08	4.94	Inf
		Abattoir number=6	8.19	45.40	22.59	Inf
		Region=Haute-Normandie	11.59	15.37	5.41	Inf
		Region=Champagne Ardennes	20.49	8.32	1.66	Inf
		Region=Basse-Normandie	7.39	38.67	21.33	Inf
		Production type=Mixed cattle	7.66	38.43	20.46	Inf
		Age=[2;3.5 years old)	26.65	86.70	13.26	Inf
		Sex=Castrated male	91.00	100	4.48	Inf
7	5,120 (1.34%)	Abattoir number=6	2.82	47.40	22.59	Inf
		Local muscular cysticercosis=1	99.77	100	1.35	Inf
		Head=1	12.87	75.39	7.87	Inf
		Heart=1	4.36	32.79	10.10	Inf
		Age=[2;3.5 years old)	3.03	29.94	13.26	31.32
		Sex=Castrated male	4.65	15.51	4.48	30.49
		Region=Basse-Normandie	2.45	38.89	21.33	28.69

^1^ Proportion of cattle that had the variable category and were in the cluster *e.g.* 15.39% of cattle that had a bronchopneumonia lesion in the whole population were in the cluster 2.

^2^ Proportion of cattle in the cluster that had the variable category *e.g.* 28.68% of cattle in the cluster 2 had a bronchopneumonia lesion.

^3^ Proportion of cattle in the global population that had the variable category *e.g.* 3.53% of the whole population had a bronchopneumonia lesion.

Categories of supplementary variables are underlined. Categories are presented according to decreasing absolute value of v-test. Inf= infinite, AMI= Ante-Mortem Inspection.

**Table 6:** Cluster description by categories of active and supplementary variables (Cluster 8 to 15)

**Cluster**	**Number of condemned animals (%)**	**Variable category**	**Cla/Mod**^**1**^	**Mod/Cla**^**2**^	**Global**^**3**^	**|v.test|**
8	9,095 (2.39%)	Other deteriorations=1	86.77	100	2.75	Inf
		Kidneys=1	5.14	41.45	19.23	Inf
		Heart=1	5.43	22.98	10.10	36.18
		Lungs=1	4.16	31.58	18.10	31.41
		Age=[8;24 months old)	3.90	33.14	20.30	28.99
9	8,993 (2.36%)	Abattoir number=10	14.42	31.80	5.20	Inf
		Region=Brittany	8.78	33.66	9.05	Inf
		Steatosis=1	83.91	100	2.81	Inf
		Kidneys=1	4.63	37.77	19.23	Inf
		Liver=1	3.07	87.87	67.52	Inf
		Production type=Dairy cattle	4.03	60.18	35.27	Inf
		Age=[5;10 years old)	3.31	52.51	37.37	29.47
		Sex=Female	2.78	82.53	69.99	27.60
		Year of slaughter=2006	4.17	22.15	12.52	25.64
10	8,461(2.22%)	Abattoir number=6	8.60	87.50	22.59	Inf
		Thymus=1	98.73	18.39	0.41	Inf
		Region=Brittany	8.14	33.16	9.05	Inf
		Nephritis=1	8.59	29.75	7.68	Inf
		Kidneys=1	6.96	60.29	19.23	Inf
		Heart=1	5.92	26.95	10.10	Inf
		Production type=Dairy cattle	4.33	68.75	35.27	Inf
		Age=[0;8 months old)	99.83	100	2.22	Inf
		Sex=Non castrated male	7.74	89.07	25.53	Inf
		Whole carcass=1	7.89	12.19	3.43	35.30
11	22,690 (5.95%)	AMI abnormality=Yes	27.82	18.28	3.91	Inf
		Arthritis=1	86.14	43.13	2.98	Inf
		Infiltration=1	83.90	49.00	3.48	Inf
		Preventive motive=1	11.88	23.93	11.99	Inf
		Udder=1	15.82	14.05	5.29	Inf
		Part of the carcass=1	46.09	99.21	12.81	Inf
		Age=≥10 years old	9.94	21.87	13.10	37.60
12	7,383 (1.94%)	Myopathy=1	94.90	100	2.04	Inf
		Part of the carcass=1	15.07	99.70	12.81	Inf
		Sex=Female	2.44	88.01	69.99	37.07
		AMI abnormality=Yes	5.30	10.70	3.91	25.32
		Abattoir number=6	2.94	34.27	22.59	23.07
		Abattoir number=8	4.47	11.40	4.94	22.34
13	8,778 (2.30%)	Abattoir number=9	6.47	36.61	13.03	Inf
		Region=Pays de la Loire	5.41	35.00	14.89	Inf
		Bronchopneumonia=1	65.30	100	3.53	Inf
		Lungs=1	12.70	99.81	18.10	Inf
		Age=[8;24 months old)	5.90	52.01	20.30	Inf
		Sex=Non castrated male	4.99	55.30	25.53	Inf
14	7,944 (2.08%)	Abattoir number=9	10.58	66.16	13.03	Inf
		Region=Poitou-Charentes	12.83	13.03	2.12	Inf
		Region=Aquitaine	19.58	17.79	1.89	Inf
		Lung emphysema=1	94.21	100	2.21	Inf
		Lungs=1	11.52	100	18.10	Inf
		Production type=Dairy cattle	4.22	71.44	35.27	Inf
		Sex=Female	2.60	87.41	69.99	37.05
		Age=[5;10 years old)	3.07	55.06	37.37	32.28
15	5,452 (1.43%)	AMI abnormality=Yes	11.05	30.21	3.91	Inf
		Meat with abnormal maturation=1	98.09	100	1.46	Inf
		Whole carcass=1	41.62	99.82	3.43	Inf
		Viscera=1	6.57	69.04	15.03	Inf
		Tongue=1	27.49	74.25	3.86	Inf
		Head=1	13.63	75.00	7.87	Inf
		Kidneys=1	5.70	76.61	19.23	Inf
		Lungs=1	5.63	71.20	18.10	Inf
		Udder=1	16.59	61.34	5.29	Inf
		Heart=1	10.86	76.71	10.10	Inf
		Sex=Female	1.80	88.08	69.99	31.91

^1^ Proportion of cattle that had the variable category and were in the cluster *e.g.* 15.39% of cattle that had a bronchopneumonia lesion in the whole population were in the cluster 2.

^2^ Proportion of cattle in the cluster that had the variable category *e.g.* 28.68% of cattle in the cluster 2 had a bronchopneumonia lesion.

^3^ Proportion of cattle in the global population that had the variable category *e.g.* 3.53% of the whole population had a bronchopneumonia lesion.

Categories of supplementary variables are underlined. Categories are presented according to decreasing absolute value of v-test. Inf= infinite, AMI= Ante-Mortem Inspection.

Clusters 1, 2, 4, 5, 7, 8, 9, 10, 12, 13, 14 and 15 contained between 1% and 2% of all the condemned cattle. Clusters 6 and 11 were larger, with respectively 4% and 6% of the condemned cattle. Cluster 3 contained almost two thirds (66%) of the condemned cattle population. No clusters were characterized by the month of slaughter (Tables 5 and 6).

The stability of the clusters according to the year of slaughter and the slaughterhouse showed high stability (more than 50%) for all clusters except clusters 5, 6 and 10 (Table 7).

**Table 7:** Cluster stability: cluster identification by the different analyses (MFA+K-means+HAC) performed by slaughterhouse and year of slaughter

	**Number of identifications**		
**Cluster**	**Analyses by slaughterhouse**^**1**^	**Analyses by year of slaughter**^**2**^	**Total **^**3 **^**(%)**
1	9	5	**14 (93)**
2	8	5	**13 (87)**
3	8	5	**13 (87)**
4	4	4	**8 (53)**
5	2	2	**4 (27)**
6	0	0	**0 (0)**
7	9	5	**14 (93)**
8	5	5	**10 (67)**
9	7	4	**11 (73)**
10	2	1	**3 (20)**
11	9	5	**14 (93)**
12	9	5	**14 (93)**
13	7	4	**11 (73)**
14	7	5	**12 (80)**
15	8	5	**13 (87)**

^1^ Number of slaughterhouses (n=10) for which the cluster has been identified.

^2^ Number of years of slaughter (n=5) for which the cluster has been identified.

^3^ Number and proportion of slaughterhouses and years (n=15) for which the cluster has been identified.

All the cattle in cluster 1 (2% of condemned cattle) presented a lesion of fecal contamination compared to 3% of the total number of cattle condemned. 82% of the cattle with a lesion of fecal contamination in the whole population were in this cluster. Condemnation of the heart and lungs also characterized this cluster with respectively 61% and 81% of the cattle from cluster 1 (Table 5).

All the cattle in the cluster 2 (2% of condemned cattle) presented a pericarditis lesion associated with heart condemnation compared to 2% of the total number of cattle condemned. 87% of cattle with a pericarditis lesion in the whole population were in this cluster. Bronchopneumonia lesion and lungs condemnation also characterized this cluster. Dairy cattle were over-represented (51% *versus* 35%) (Table 5).

Cluster 3 was the largest cluster (66% of the population). This cluster was characterized by liver condemnation and by lesions associated with the liver such as macular telangiectasia, liver fluke, sclerosis, and abscess. Beef cattle, female and age categories over 5 years of age also characterized this cluster (Table 5).

All cattle in cluster 4 (2% of condemned cattle) presented with a peritonitis lesion. “Part of the carcass” was a condemned portion that also characterized this cluster (54% of cattle in the cluster *versus* 13% of cattle in the whole dataset). This cluster was also characterized by female and dairy cattle. Abscess concerned 43% of cattle in the cluster *versus* 24% in the whole population (Table 5).

Cluster 5 (2% of condemned cattle) was characterized by whole carcass condemnation for 85% of cattle *versus* 3% in the whole dataset. The other condemned portions that characterized this cluster were viscera, tongue, kidneys, heart, liver, head, lungs, and udder. This is linked to the fact that the entire cattle carcass is defined as presented in Figure 2, thus the condemnation of the entire animal means the whole carcass, offal, head and udder. Pleurisy, congestion and peritonitis characterized this cluster. In this cluster, 21% of cattle had presented at least one symptom during ante-mortem inspection whereas only 4% of the whole population of the study had one (Table 5).

Cluster 6 (4% of condemned cattle) was only characterized by demographic variable levels: castrated male, mixed cattle and cattle from 2 to 3.5 years of age. All cattle in this cluster were castrated males and 91% of castrated males in the whole population were in this cluster (Table 5).

All cattle in cluster 7 (1% of condemned cattle) presented local muscular cysticercosis lesions. 99.8% of the whole population with a lesion of local muscular cysticercosis was in this cluster. This cluster was also characterized by condemnation of head and tongue. Castrated males and cattle from 2 to 3.5 years of age characterized this cluster (Table 5).

All cattle in cluster 8 (2% of condemned cattle) presented a lesion called “other deteriorations”. Additionally, 87% of the whole population with this lesion was in this cluster (Table 6).

All cattle in cluster 9 (2% of condemned cattle) presented steatosis lesions. 84% of the whole population found to have this lesion was in this cluster. The other characteristic levels were kidneys and liver condemnation, dairy cattle and the 5-to-10-year age group (Table 6).

All cattle in cluster 10 (2% of condemned cattle) were under 8 months of age. 99.8% of the whole population of cattle under 8 months of age was in this cluster. Nephritis characterized this cluster with 30% of cattle concerned in this cluster *versus* 8% in the whole population. Thymus, kidneys, heart and whole carcass condemnation characterized the cluster. Dairy cattle and non-castrated males were more frequent in cluster 10 than in the whole population (Table 6).

Cluster 11 (6% of condemned cattle) was characterized by the condemnation of part of the carcass with 99% of the cattle concerned in the cluster *versus* 13% in the whole population. Arthritis and inflammation characterized the cluster with respectively 43% and 49% of cattle in the cluster concerned. In this cluster 18% of cattle had presented at least one symptom during ante-mortem inspection whereas only 4% had in the whole population of the study (Table 6).

All cattle in cluster 12 (2% of condemned cattle) had a lesion of myopathy and 95% of the whole cattle population affected by myopathy was in this cluster. 99.7% of cattle in this cluster had a condemnation of part of the carcass. This cluster was also characterized by female gender (88% within the cluster *versus* 70% in the whole population) (Table 6).

All cattle in cluster 13 (2% of condemned cattle) had a bronchopneumonia lesion and 65% of the whole cattle population affected by bronchopneumonia was in this cluster. This cluster was also characterized by the condemnation of lungs with 99.8% of cattle in the cluster concerned *versus* 18% in the whole population. Non-castrated males and cattle from 8 to 24 months of age characterized this cluster (Table 6).

All cattle in cluster 14 (2% of condemned cattle) had a lung emphysema lesion associated with condemned lungs and 94% of the whole cattle population affected by lung emphysema was in this cluster. Female, dairy cattle and cattle from 5 to 10 years of age characterized this cluster (Table 6).

All cattle in cluster 15 (1% of condemned cattle) presented meat with abnormal maturation and 98% of the whole cattle population affected by abnormal meat maturation was in this cluster. This cluster was characterized by whole carcass condemnation (99.8% of cattle in this cluster had their whole carcass condemned *versus* 3% in the whole population). For the same reason as for cluster 5, viscera, tongue, head, kidneys, lungs, udder and heart condemnation also characterized this cluster. Additionally, 30% of cattle in this cluster had presented at least one symptom during ante-mortem inspection whereas this was the case for only 4% of the whole population present in the study. Females also characterized this cluster (Table 6).

## Discussion

From the perspective of using meat inspection data for syndromic surveillance purposes, the objective of this study was to define syndromes through a statistical approach. MFA in combination with clustering methods was performed to determine a typology of cattle that had at least one condemned carcass portion based on meat inspection data collected in ten slaughterhouses. Results led to 15 clusters characterized by reasons for condemnation, condemned portions and demographic parameters.

### Material

The data available for each slaughterhouse did not cover the same period. However, the total amount of data (381,186 condemned cattle) was considered sufficient to define the main types of groups of lesions. The stability of the typology according to year of slaughter and slaughterhouse demonstrated the low impact of year and slaughterhouse for 12 out of the 15 clusters (Table 7). The interpretation of the three clusters with low stability is discussed below.

Among the 381,186 cattle with at least one portion of the carcass condemned, there were only 25,031 different combinations of observed variable levels. This highlighted the fact that condemned cattle frequently had matching values for the active variables (sex, age category, production type, condemned portion and reason for condemnation). This could be explained by the fact that i) data were grouped for MFA analysis such as age in age categories and some of the reasons for condemnation, ii) official inspectors could not register more than one reason for condemnation for each condemned portion which reduced the variability of reasons for condemnation for each animal, iii) cattle arriving at the slaughterhouses were usually in good health as it is expected by European regulation, so the diversity of lesions should be lower than in the general cattle population.

### Combination of principal component method and hybrid clustering

The results of a principal component method would have been too complex for a direct extraction of a typology of condemned cattle due to the large number of variable levels involved. Using directly a clustering method on both demographic and condemnation data was not feasible due to the issue of distance definition. Moreover, conducting a clustering analysis on a large number of both individuals and categorical variables is challenging.

To face this issue, combining principal component method i.e. MFA, and hybrid clustering i.e. K-means and HAC, is a relevant analytical approach. Principal component method such as MFA allowed the definition of a distance between condemned cattle based on several sets of categorical variables (demographic and condemnation data) through the computation of the Euclidean distance from the individual principal coordinates from MFA. The hybrid clustering method allowed the use of HAC despite the large number of units using K-means clustering method as a first step and performing HAC on the K-means centroids of clusters.

### From statistical cluster to syndrome definition

Cluster 1 and 8 can be linked to the quality of the slaughtering process. Indeed, cluster 1 was characterized by fecal contamination of the heart or lungs. These lesions are due to a failure in the slaughtering process especially during the evisceration stage. The “other deteriorations” that characterized cluster 8 grouped together different lesions that revealed issues in the slaughtering process. No interpretation has been found for the fact that cattle from 8 to 24 months of age characterized cluster 8.

Cluster 2, 9 and 14 can be linked to management practice issues, such as feeding, and to diseases of economic importance to farmers. The combination of lesions found in cluster 2 (i.e. pericarditis, bronchopneumonia) could be the result of cattle swallowing sharp foreign bodies (metallic or not), causing traumatic reticuloperitonitis and pericarditis. This condition is more frequent in dairy cattle and has been shown to be a culling criteria [21-23]. Both traumatic pericarditis and bronchopneumonia could be linked to management practices such as feeding (for traumatic pericarditis) [24]. Characteristics of cluster 9 fit the definition of the well-known fatty liver syndrome in dairy cattle. This syndrome occurs in high-producing dairy cattle when overfeeding in the dry period results in overfat cows at calving [25-27]. Fatty liver has a high economic impact on farmers as it is linked to decreased health status and reproductive performance [26]. Emphysema, lesion that characterized cluster 14, is commonly associated with hypersensitivity pneumonia also called “farmer’s lung”. This disease affects mainly adult dairy cattle, which is consistent with the description of this cluster [28,29]. Animals develop this condition as a result of exposure to hay with high moisture content. It has economic consequences due to the resulting decrease in milk production.

Cluster 3 and 4 were characterized by lesions linked to diseases of economic importance to farmers. Cluster 3 dealt with chronic liver lesions, common in old cows. These conditions have a direct economic impact on farmers due to liver condemnation and an indirect impact due to the consequences of these conditions, especially liver flukes on production levels [30,31]. Brown et al. [32] showed an association between liver abnormalities such as telangiectasis, distoma, abscesses, cirrhosis and subsequent changes in carcass characteristics which ultimately resulted in a loss of carcass value. Abscesses and liver flukes were the most frequent lesions observed in the condemned population, which could explain the large size of this cluster (Tables 3, 5 and 6). For cluster 4, as whole carcass was not characteristic of this cluster, we could infer that the peritonitis lesions that characterized this cluster were chronic lesions. Chronic peritonitis can be linked to different kinds of conditions such as traumatic reticulo-peritonitis or the consequences of dystocia [33]. Abscesses are commonly linked with reticulo-peritonitis [34]. This type of condemnation has a direct economic impact on farmers through the condemnation of the related portion of the carcass but also reveals a previous major cattle condition that had probably caused a decrease in production.

Cluster 7 can be linked to a public health issue as it was characterized by cysticercosis, a zoonotic disease. A study conducted on cattle slaughtered in France in 2010 showed that cysticercosis lesions were more frequent in cattle from 2 to 4 years of age which is consistent with the description of this cluster [35]. Moreover this cluster was also characterized by condemnation of the head and tongue, which are the portions usually affected by cysticercosis [36-38].

Cluster 11 can be linked to an animal health and welfare issue, with an important economic impact on farmers. Indeed, this cluster was characterized by arthritis, a lesion of pathological significance [39]. Arthritis is commonly caused by i) direct trauma or penetration by a contaminated foreign body (primary arthritis), ii) spread of pathogens from an adjacent localized area or from systemic spread from another area in the animal (secondary and tertiary arthritis). Pathogens commonly isolated in arthritis include *E. coli*, *Staphylococcus* and *Streptococcus spp*[27]. Arthritis has a direct financial impact for farmers through condemnation and also because affected cattle present an abrupt drop in milk yield [27]. This last point explains that arthritis is a culling criteria [22]. These common causes of arthritis and the proportion of ante-mortem anomalies could suggest that this cluster deals with both animal welfare and animal health issues.

Cluster 12 can be linked to an animal welfare issue and management practices. Indeed, the myopathy lesion, that characterized this cluster, is used at slaughterhouses to describe muscular lesions due to previous trauma also known as muscle crush syndrome [28].

Cluster 13 can be linked to animal health issues and management practices. As bronchopneumonia was characteristic of cluster 13 but not associated with the whole carcass condemnation, we could hypothesize that this cluster deals with chronic bronchopneumonia lesions. Bronchopneumonia is caused by numerous combinations of ubiquitous infectious agents that produce disease mostly when host defenses are lowered by stress, nutritional deficiencies or respiratory virus infections [28].

Cluster 15 can be linked to both animal welfare, especially transport management, and economic impact on farmers. Indeed, this cluster was characterized by whole carcass condemnation for abnormal maturation of the meat. The major abnormal maturation of meat that results in whole carcass condemnation is dark, firm, dry meat (DFD). Such meat looks abnormal for consumers and is condemned for organoleptic reasons with a significant economic impact on farmers. Pre-slaughter handling, including prolonged transport and emotional stress due to grouping of animals, was recognized as the main risk factor of DFD meat [40,41].

### Clusters defined as not stable

Clusters 5, 6 and 10 were considered as not stable according to year of slaughter and slaughterhouses.

Cluster 5 represented animals with acute inflammation of serous membranes (i.e. pleurisy, peritonitis, congestion). Indeed, as whole carcass condemnation was characteristic of this cluster, we could interpret that the pleurisy, congestion and peritonitis lesions were acute lesions. The direct economic impact on farmers is huge because of the whole carcass condemnation, which reveals a recent issue of animal health in the herd that should be investigated.

We hypothesized that cluster 6 grouped almost all the castrated males together (91% of all castrated males were in this cluster and 100% of cattle in this cluster were castrated males) without identifying any characteristic reasons for condemnation because castrated males had similar lesions to the whole population of the study.

All cattle in cluster 10 were under 8 months of age, which explains that thymus, an organ which disappears in older animals, was more frequent in this cluster than in the whole population. Almost all cattle under 8 months old were in this cluster (99.8%). Nephritis is a common lesion in calves frequently due to *E. coli*[29]. The condemned portions (heart, kidneys, thymus) and condemnation lesion (nephritis) that characterized this cluster could also be caused by bronchopneumonia affecting calves that secondarily induces lesions on heart, kidneys and thymus when it is untreated [29].

### Cluster homogeneity

Some reasons for condemnation were highly differential as all cattle in the cluster had the same reason for condemnation: fecal contamination (cluster 1), pericarditis (cluster 2), peritonitis (cluster 4), cysticercosis (cluster 7), other deteriorations (cluster 8), steatosis (cluster 9), myopathy (cluster 12), bronchopneumonia (cluster 13), lungs emphysema (cluster 14), and DFD meat (cluster 15).

As presented previously, two clusters were mainly defined by a demographic characteristic: cluster 10 by cattle under 8 months old and cluster 6 by non-castrated males.

This statistical approach allowed the identification of homogeneous groups of cattle according to reasons for condemnation and/or demographic characteristics.

### Links between ante-mortem and post-mortem inspection

This analysis showed that presence of clinical signs during ante-mortem inspection characterized clusters 5, 11 and 15. These clusters were characterized respectively by acute inflammation of serous membranes, arthritis and DFD Meat. This led us to believe that ante-mortem inspection could be particularly relevant to detect these conditions. This result was expected for acute inflammation of serous membranes and arthritis but surprising for DFD meat.

### The added value of the statistical approach

Considering the large number of reasons for condemnation, condemnation portions and demographic data, it is not feasible to monitor individually each potential combination. Using an experts group as a first step to deal with this issue would be time consuming to reach a consensus, if a consensus could be reached.

This study showed that a statistical descriptive approach could help defining groups of lesions of biological interest based on the reality of the existing lesions even if the data are numerous and complex. Indeed, this typology provided meaningful ideas on groups of reasons for condemnation that could be interesting to monitor and that would probably not have been spontaneously defined by a group of experts. The advantages of this statistical approach are i) to define a typology of lesions profiles based on already existing reasons for condemnation, ii) to aid in grouping together reasons for condemnation from a list of possible reasons for condemnation for which a consensus among experts could be difficult to reach without this initial descriptive approach, iii) to assign each animal into a single syndrome which could enable the detection of changes in trends for the percentages of each syndrome in the slaughtered population, in order to detect emerging unknown diseases. Indeed, each disease is characterized by group(s) of lesions and a specific susceptible population characterized by demographic criteria; it seems thus relevant to assume that most cattle affected with an emerging disease will share similar reasons for condemnation and demographic characteristics. Using the statistical syndrome definition, each new condemned animal will be attributed to a cluster already defined by determining its MFA-derived representation and the cluster it belongs to. It is thus probable that most of these affected cattle will be attributed to the same cluster, making its proportion abnormally increasing.

The description of this typology showed that slaughterhouse data can be relevant not only for animal health or public health surveillance but also for animal welfare assessment, evaluation of the quality of the slaughtering process, management issues, and evaluation of the economic impact on farmers. Indeed, among the 15 clusters, three were linked to issues that occur outside the farm i.e. animal welfare during transport between farm and slaughterhouse (cluster 15) and quality of the slaughtering process (clusters 1 and 8).

In order to use these data for these different objectives, relevant indicators have to be defined for monitoring. Additional file 3 presents, for each stable cluster, a brief interpretation of the cluster and recommendations in terms of level of analysis for indicators that could be built for surveillance purposes.

Depending on the field of interest, the indicator could be analyzed at different levels. To detect problems in the quality of the slaughtering process an indicator at the slaughterhouse level is relevant. It could be monitored in real time to be able to conduct corrective measures early; and it could also be used as a quarterly or annual indicator to classify slaughterhouses in order to perform risk-based veterinary controls in slaughterhouses.

For management practices, economic impact on farmers, animal health and animal welfare issues, indicators at the farm level could be relevant for farmers so as to be able to identify gaps in their practices and take corrective measures to decrease economic impact. On the other hand, an annual indicator at the herd level for animal health and welfare issues could be relevant to organize risk-based official controls of herds.

## Conclusion

The typology of the 15 groups of lesions obtained highlighted 12 frequent groups of lesions which were stable regardless of the slaughterhouses or years of slaughter studied. The application of slaughterhouse data and this typology for epidemiology surveillance could be done using two different approaches:

1) Define and monitor indicators in real time:

a. To detect an abnormal change of a specific syndrome defined by a group of experts based or not on the statistical definition of syndromes presented in the present study, *e.g.* definition of groups of reasons for condemnation and portions to monitor reticulo-peritonitis. Slaughterhouse data in particular are an added value for diseases for which post-mortem lesions are more specific than clinical symptoms (*e.g.* fatty liver syndrome). Space-time clustering of cattle affected by these syndromes could be performed to implement a syndromic surveillance system [42].

a. To monitor specific diseases for which data are only available at the slaughterhouse such as fascioliasis or cysticercosis. If a database such as the *Nergal-Abattoir* database was implemented in all the slaughterhouses of a country, it could allow the implementation of real-time traditional surveillance with exhaustive data to detect outbreaks and analyze trends of these diseases.

a. To detect an emerging disease by monitoring the percentage of cattle in each cluster defined statistically to detect changes in trends. Each new slaughtered animal will be attributed to the defined syndromes by determining its MFA-derived representation and the cluster it belongs to. Real-time analysis of the syndromic time series with anomaly detection algorithms could then be performed to implement a syndromic surveillance system.

2) Define monthly, quarterly or annual indicators:

a. At herd level for farmers, so as to highlight poor practices and then enable them to implement corrective actions.

a. At herd or *département* level, for veterinary services to focus their controls on high risk herds/areas.

a. At slaughterhouse level, for monitoring the quality of the slaughtering process.

Our results showed the various implications of slaughterhouse data for surveillance as well as for herd management and optimization of veterinary services controls. This should encourage increasing use of meat inspection data. Even though these datasets are complex, our study showed that it is possible to define groups of underlying reasons for condemnation and portions, taking into account both demographic and lesion data on slaughtered cattle.

## Abbreviations

AMI: Ante-mortem inspection; DFD: Dark Firm Dry; HAC: Hierarchical Ascendant Clustering; MFA: Multiple Factorial Analysis; PMI: Post-mortem inspection

## Competing interests

The authors declare that they have no competing interests.

## Authors’ contributions

CéD performed the statistical analysis and drafted the manuscript. EM was involved in the analysis of data. XM was involved in data acquisition through database design. JLV made substantial contributions to data design. PH, ChD, DC and EG were involved in the interpretation of data, and critical revising the manuscript for important intellectual content. All authors read and approved the final manuscript.

## Supplementary Material

Additional file 1 Additional information on the statistical method used to define the typology of condemned bovine. Click here for file

Additional file 2 Description of cattle condemned according to last farm location, year and month of slaughter, and ante-mortem inspection abnormality. Click here for file

Additional file 3 Proposal of the potential use of each cluster to define indicators for future statistical analysis. Click here for file
