# Supplementary material for: Defining syndromes using cattle meat inspection data for syndromic surveillance purposes: a statistical approach with the 2005–2010 data from ten French slaughterhouses
Source: BMC Vet Res. 2013 Apr 30;9:88. doi: 10.1186/1746-6148-9-88 (PMC3681570; doi:10.1186/1746-6148-9-88)
Supplement: Additional file 1 — Additional information on the statistical method used to define the typology of condemned bovine. [file 1746-6148-9-88-S1.docx]

Additional file 1: Additional information on the statistical method used to define the typology of condemned bovine.

**Multiple Factorial Analysis (MFA)**

MFA is a principal component method as well as Principal Component Analysis (PCA) for quantitative variables, Multiple Component Analysis (MCA) for categorical variables and Component Analysis (CA) for frequency variables. MFA should not be confused with factor analysis. The principle of these principal component methods is to reduce the dimensionality of large multivariate datasets replacing the n original variables (active or observed variables) by p uncorrelated derived variables (principal components or factors) obtained by linear combination of active variables. Each factor is orthogonal to each other (i.e. defined so that it captures the variance not explained by the previous factor) and is defined by its eigenvalue which indicates the inertia (i.e. variance) of the data it represents. The importance of a principal component is thus reflected by its variance or proportion of the total variance. One of the outputs of principal component methods is the principal coordinates of each unit in the factorial space defined by factorial axes. It allows the computation of a distance between two units.

The particularity of MFA is to compute a global distance between units based on several sets of variables and balancing the influence of each set of variables in this computation. Each set of variables can be of one of these three types: categorical, quantitative or frequency variables.

In this study we only used sets of categorical variables thus performing MFA induced a distance between units corresponding to a weighted sum of the separate distances induced by Multiple Component Analysis (MCA) performed on each set of variables.

MFA principal coordinates of each condemned bovine were used as input for clustering methods. Last factorial axes are commonly considered as non informative, just conveying “noise”, that is why only the first axes ensuring 95% of the total variance are commonly considered.

**Hybrid clustering: K-means and Hierarchical Ascendant Clustering**

Several clustering methods can be used such as partitioning methods or hierarchical methods, each of them having strengths and weaknesses. Hybrid clustering consists in combining several clustering methods to take advantage of their strengths and limit their weaknesses. It is commonly used to deal with large datasets.

All clustering methods seek to divide observations into similar groups involving a definition of similarity through a distance definition. In this study, a classical Euclidean distance was computed from the principal coordinates of MFA.

Hierarchical Ascendant Clustering (HAC) is a hierarchical method, producing a nested sequence of clusters, i.e. a hierarchy of clusters. Each cutting level gives a different sequence of clusters. HAC is a bottom up approach where each individual starts in its own cluster, and pairs of clusters are then merged as one moves up the hierarchy. A linkage criterion i.e. definition of the distance between sets of observations, is needed. Ward’s criterion is a common linkage criterion which minimizes intra-cluster variance and maximizes inter-cluster variance. The weakness of HAC is its high computation and storage requirement that makes it difficult to implement on large datasets. Hybrid clustering is an answer to this issue using partitioning method as a first step for HAC. Partitioning methods seek to optimally divide objects into a fixed number of clusters, and are particularly efficient for clustering large datasets.

K-means is the most popular partitioning method. Its algorithm seeks to find *n* clusters that minimize the sum of squared Euclidean distances between each observation and its respective cluster mean through an iterative alternating fitting process between two steps: 1) assign each observation to the cluster with the nearest center 2) update each cluster center as the mean for points in that cluster. The process continues until no further changes occur in the clusters. The *n* number of clusters needs to be defined as an input. This number was defined in our study as the number of distinct coordinates in the 30-dimensional factorial space of MFA.

K-means was used as a first step to reduce the data to a limited number of homogeneous clusters so as to be able to perform a HAC on the clusters centers.

After choosing the cutting level of HAC, the clusters obtained were consolidated by performing the K-means algorithm.
