# Supplementary material for: Defining syndromes using cattle meat inspection data for syndromic surveillance purposes: a statistical approach with the 2005–2010 data from ten French slaughterhouses
Source: BMC Vet Res. 2013 Apr 30;9:88. doi: 10.1186/1746-6148-9-88 (PMC3681570; doi:10.1186/1746-6148-9-88)
Supplement: Additional file 2 — Description of cattle condemned according to last farm location, year and month of slaughter, and ante-mortem inspection abnormality. [file 1746-6148-9-88-S2.docx]

Additional file 2: Description of cattle condemned according to last farm location, year and month of slaughter, and ante-mortem inspection abnormality.

Condemned cattle = cattle with at least one portion of the carcass condemned.

|  | Number of cattle condemned | Percentage of condemnations (%) |
| --- | --- | --- |
| Farm location |  |  |
| Basse-Normandie | 81,308 | 21.33 |
| Pays de la Loire | 56,777 | 14.89 |
| Auvergne | 36,962 | 9.70 |
| Brittany | 34,483 | 9.05 |
| Haute-Normandie | 20,606 | 5.41 |
| Burgundy | 20,214 | 5.30 |
| Rhône-Alpes | 19,929 | 5.23 |
| Centre | 17,245 | 4.52 |
| Lorraine | 16,294 | 4.27 |
| Nord-Pas de Calais | 13,891 | 3.64 |
| Picardie | 13,272 | 3.48 |
| Franche-Comté | 11,194 | 2.94 |
| Other regions | 9,072 | 2.37 |
| Limousin | 8,345 | 2.19 |
| Poitou-Charentes | 8,066 | 2.12 |
| Aquitaine | 7,217 | 1.89 |
| Champagne Ardennes | 6,311 | 1.66 |
| Year of slaughter |  |  |
| 2005 | 14,221 | 3.73 |
| 2006 | 47,733 | 12.52 |
| 2007 | 95,766 | 25.12 |
| 2008 | 100,628 | 26.4 |
| 2009 | 86,356 | 22.65 |
| 2010 | 36,482 | 9.57 |
| Month of slaughter |  |  |
| January | 30,081 | 7.89 |
| February | 25,019 | 6.56 |
| March | 27,994 | 7.34 |
| April | 27,952 | 7.33 |
| May | 26,275 | 6.89 |
| June | 31,432 | 8.25 |
| July | 35,631 | 9.35 |
| August | 38,350 | 10.06 |
| September | 35,663 | 9.36 |
| October | 36,657 | 9.62 |
| November | 33,939 | 8.9 |
| December | 32,193 | 8.45 |
| Ante-mortem inspection abnormality |  |  |
| Yes | 14,909 | 3.91 |
| No | 366,277 | 96.09 |
