# Supplementary material for: Defining syndromes using cattle meat inspection data for syndromic surveillance purposes: a statistical approach with the 2005–2010 data from ten French slaughterhouses
Source: BMC Vet Res. 2013 Apr 30;9:88. doi: 10.1186/1746-6148-9-88 (PMC3681570; doi:10.1186/1746-6148-9-88)
Supplement: Additional file 3 — Proposal of the potential use of each cluster to define indicators for future statistical analysis. [file 1746-6148-9-88-S3.docx]

Additional file 3: Proposal of the potential use of each cluster to define indicators for future statistical analysis.

Only “stable” clusters are presented.

| Cluster | Brief description | Field | Level of analysis of the indicator |
| --- | --- | --- | --- |
| 1 | Faecal contamination of heart or lungs linked with failure during evisceration step | Quality of slaughtering process | Slaughterhouse through a quality indicator |
| 8 | Alterations linked to failure in the slaughtering process |  |  |
| 2 | Pericarditis and bronchopneumonia which could be linked to traumatic reticulo-pericarditis | Management practices such as feeding | Herd level through a quarterly or annual indicator for farmers and veterinary services (inspection on herds) |
| 9 | Fatty liver syndrome |  |  |
| 14 | Lung emphysema : farmer’s lung |  |  |
| 3 | Chronic liver lesions | Management practices | Herd level through a quarterly or annual indicator |
| 4 | Chronic peritonitis lesions which could be linked to previous infection |  |  |
| 11 | Arthritis | Animal health  Animal welfare | Herd level through a quarterly or annual indicator for farmers and veterinary services (controls in herds) |
| 12 | Myopathy (muscle crush syndrome) | Animal welfare  Management practices |  |
| 13 | Bronchopneumonia | Animal health  Management practices |  |
| 15 | DFD meat | Animal welfare (transport management) | Herd level and transporters level through a quarterly or annual indicator for farmers and veterinary services (controls in herds and transporters) |
| 7 | Cysticercosis | Public health (zoonotic disease) | Space-time clustering  Daily monitoring  Classical surveillance |
| All 1-15 | Frequency of  each cluster | Emerging diseases | Detection of trend changes in syndromes to detect unknown emerging diseases by space-time modeling of the 15 clusters |
